# Cardiac Arrhythmias as Manifestations of Nanopathies: An Emerging View

**DOI:** 10.3389/fphys.2018.01228

**Published:** 2018-09-04

**Authors:** Przemysław B. Radwański, Christopher N. Johnson, Sándor Györke, Rengasayee Veeraraghavan

**Affiliations:** ^1^Bob and Corinne Frick Center for Heart Failure and Arrhythmia, The Ohio State University Wexner Medical Center, Columbus, OH, United States; ^2^Dorothy M. Davis Heart and Lung Research Institute, College of Medicine, The Ohio State University Wexner Medical Center, Columbus, OH, United States; ^3^Department of Physiology and Cell Biology, College of Medicine, The Ohio State University, Columbus, OH, United States; ^4^Division of Pharmacy Practice and Science, College of Pharmacy, The Ohio State University, Columbus, OH, United States; ^5^Vanderbilt Center for Arrhythmia Research and Therapeutics, Nashville, TN, United States; ^6^Department of Biomedical Engineering, The Ohio State University, Columbus, OH, United States

**Keywords:** nanopathies, arrhythmias, cardiac, sodium channels, sodium calcium exchanger, RyR2

## Abstract

A nanodomain is a collection of proteins localized within a specialized, nanoscale structural environment, which can serve as the functional unit of macroscopic physiologic processes. We are beginning to recognize the key roles of cardiomyocyte nanodomains in essential processes of cardiac physiology such as electrical impulse propagation and excitation–contraction coupling (ECC). There is growing appreciation of nanodomain dysfunction, i.e., nanopathy, as a mechanistic driver of life-threatening arrhythmias in a variety of pathologies. Here, we offer an overview of current research on the role of nanodomains in cardiac physiology with particular emphasis on: (1) sodium channel-rich nanodomains within the intercalated disk that participate in cell-to-cell electrical coupling and (2) dyadic nanodomains located along transverse tubules that participate in ECC. The beat to beat function of cardiomyocytes involves three phases: the action potential, the calcium transient, and mechanical contraction/relaxation. In all these phases, cell-wide function results from the aggregation of the stochastic function of individual proteins. While it has long been known that proteins that exist in close proximity influence each other’s function, it is increasingly appreciated that there exist nanoscale structures that act as functional units of cardiac biophysical phenomena. Termed nanodomains, these structures are collections of proteins, localized within specialized nanoscale structural environments. The nano-environments enable the generation of localized electrical and/or chemical gradients, thereby conferring unique functional properties to these units. Thus, the function of a nanodomain is determined by its protein constituents as well as their local structural environment, adding an additional layer of complexity to cardiac biology and biophysics. However, with the emergence of experimental techniques that allow direct investigation of structure and function at the nanoscale, our understanding of cardiac physiology and pathophysiology at these scales is rapidly advancing. Here, we will discuss the structure and functions of multiple cardiomyocyte nanodomains, and novel strategies that target them for the treatment of cardiac arrhythmias.

## The Nano-Machinery of Cardiac Electrical Excitation

For over a century, it has been recognized that cardiac myocytes come into close contact at the intercalated disk (ID), where adjacent cells are only nanometers apart. By the mid-20^th^ century, gap junctions (GJs) were identified as specialized structures within the ID, which afford electrochemical coupling between cells (Sjostrand and Andersson, 1954; Barr et al., 1965). The closest apposition between neighboring myocytes occurs within these GJs, with as little as 2 nm separating the cells. Despite this, GJs themselves do not constitute a functional nanodomain – they afford direct cytosolic continuity between coupled cells, and since neither cytosolic compartment is a restricted environment, the effects unique to nanoscale compartments do not come into play. Thus, cardiac conduction, the cell-to-cell spread of electrical excitation through the heart, was thought a relatively simple process with cardiac voltage-gated sodium (Na^+^) channels (Na_V_1.5) affording excitability, and GJs providing cell-to-cell electrical coupling (Kleber and Rudy, 2004).

This electrotonic view of cardiac conduction allowed the formalism of cable theory to be applied to cardiac conduction, and adequately explained experimental observations for several decades (Kleber and Rudy, 2004). However, findings have accumulated, that are not well-explained by this model. Transgenic mice with reduced Cx43 expression displayed significant conduction slowing in some studies (Guerrero et al., 1997; Eloff et al., 2001; Gutstein et al., 2001), but not in others (Morley et al., 1999; Vaidya et al., 2001; Thomas et al., 2003; Beauchamp et al., 2004; Danik et al., 2004; van Rijen et al., 2004; Stein et al., 2009). Likewise, GJ remodeling and reduced Cx43 expression correlated with arrhythmogenic conduction slowing in one pacing-induced canine heart failure model (Poelzing et al., 2004), but preceded it in another (Akar et al., 2007). Another discrepant finding stemmed from investigations of the electrophysiological impact of cardiac interstitial edema (fluid accumulation in the extracellular space): per the electrotonic model, an increase interstitial volume would lower extracellular resistance, and consequently, increase conduction velocity (Spach et al., 2004; Plonsey and Barr, 2007). However, experimental measurements in guinea pig ventricles revealed conduction slowing during to interstitial edema (Veeraraghavan et al., 2012, 2015, 2016) and edema was linked to reversible conduction block in patients undergoing ablation for the treatment of atrial fibrillation (AF) (Arujuna et al., 2012). These findings prompted speculation that non-electrotonic mechanisms of intercellular communication may play a role in the heart.

**FIGURE 1:**
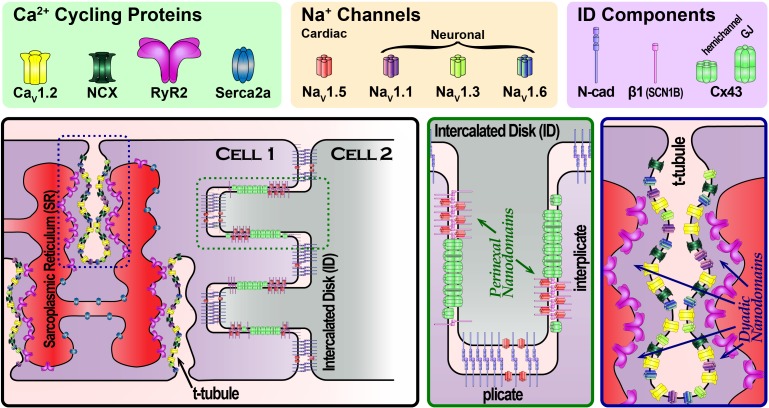
Schematic cartoon depicting the organization of Na^+^ channels in different parts of the cardiac myocyte, highlighting Na^+^ channel-rich nanodomains within the intercalated disk (green dashed box) and t-tubules (blue dashed box). Zoomed in views of these nanodomains are provided in the green and blue boxed panels.

Theoretical studies had long raised the possibility of an alternate mode of intercellular coupling (Pertsov and Medvinskii, 1976; Sperelakis and Mann, 1977; Kucera et al., 2002; Sperelakis and McConnell, 2002; Copene and Keener, 2008; Mori et al., 2008). Dubbed ephaptic coupling, this mechanism envisions cells communicating via local extracellular electrochemical transients (ion accumulation/depletion). The aforementioned models suggest that a cardiac ephapse (a functional nanodomain capable of supporting ephaptic coupling) would require Na_V_1.5-rich membranes from neighboring cells, separated by a very narrow extracellular cleft (≤30 nm) (Veeraraghavan et al., 2014a,b). Whereas channels located at the lateral sarcolemma would face a large bulk of extracellular fluid with a practically inexhaustible supply of ions, channels facing narrow extracellular clefts would have a limited supply of ions. In the latter case, ion channel activity could mount local extracellular electrochemical transients, in turn altering the local transmembrane potential. In this context, early results demonstrating that Na_V_1.5 channels are enriched at the ID (Maier et al., 2004; Petitprez et al., 2011) prompted speculation that functional nanodomains capable of supporting ephaptic coupling, i.e., ephapses, may exist within the ID. Until recently, the 250–350 nm resolution limit imposed on confocal microscopy by diffraction had precluded precise localization of Na_V_1.5 within the ID. However, this restriction was removed by the advent of super-resolution microscopy techniques with resolutions extending down to 20 nm. Work conducted by the Gourdie (Rhett et al., 2012; Veeraraghavan et al., 2013, 2015; Veeraraghavan and Gourdie, 2016) and Delmar (Agullo-Pascual et al., 2013, 2014; Leo-Macias et al., 2016) labs using super-resolution techniques identified Na_V_1.5 enrichment within specific regions of the ID (**Figure 1**). One sub-population of ID-localized Na_V_1.5 was located at the perinexus, a specialized nanodomain located at the periphery of Cx43 GJs (Rhett et al., 2011; Rhett and Gourdie, 2012). A second localized to N-cadherin-rich plicate regions of the ID, where mechanical junctions are concentrated. Electron microscopy studies revealed disparate ultrastructural properties at these sites: within the perinexus, adjacent cell membranes were no more than 10–15 nm apart (Veeraraghavan et al., 2015), whereas at N-cadherin-rich mechanical junctions, intermembrane spacing was as high as 60–75 nm (Leo-Macias et al., 2015, 2016). The latter significantly exceeds the theoretically derived ≤ 30 nm intermembrane spacing limit for ephaptic coupling; however, the properties of the perinexus may enable it to function as an ephapse.

Providing functional support for the ephaptic coupling hypothesis, the Poelzing and Gourdie groups demonstrated that acute interstitial edema selectively disrupted perinexal ultrastructure without altering other ID sites, and precipitated arrhythmogenic conduction slowing (Veeraraghavan et al., 2012, 2015, 2016). Additionally, perinexal disruption increased the dependence of conduction on GJ coupling and vice versa, suggesting that both modes of intercellular coupling operate in tandem (Veeraraghavan et al., 2012). Notably, in a setting of perinexal disruption, inhibition of channels enriched at perinexal sites – Na_V_1.5, and inward-rectifier potassium channels (K_ir_2.1) – resulted in anisotropic changes in conduction velocity, preferentially affecting transverse conduction (Veeraraghavan et al., 2015, 2016). In contrast, modulating ionic currents under normal conditions does not produce direction-specific impact. Keener and colleagues demonstrated that these experimental results could be predicted by a computational model incorporating both ephaptic and electrotonic coupling, but not by a purely electrotonic model (Lin and Keener, 2010, 2014; Veeraraghavan et al., 2015, 2016). Recently, Hichri and colleagues provided experimental and *in silico* evidence that the behavior of ID-localized Na_V_1.5 is modulated by their clustering, location within the ID (relative to the bulk interstitium), and the trans-alignment of these clusters (Hichri et al., 2017). In sum, these results illustrate macroscopic physiologic phenomena driven by the behavior of functional nanodomains, which can only be understood through knowledge of structure–function relationships at the nanodomain level.

In the larger context, the results highlighting the perinexus as a functional nanodomain involved in cardiac cell-to-cell communication pose the question: is the ID home to other functional nanodomains? The likelihood is high, given that the ID contains other sites enriched with Na_V_1.5 (Milstein et al., 2012; Veeraraghavan et al., 2015; Leo-Macias et al., 2016) and K_ir_2.1 (Milstein et al., 2012; Veeraraghavan et al., 2016; Ponce-Balbuena et al., 2018) as well as other ion channel species such as ATP-sensitive K^+^ channels (K_ir_6.2) (Hong et al., 2012). Notably, extracellular sodium has been shown to strongly modulate K_ir_2.1 function (Ishihara, 2018), hinting at the potential for complex interrelationships between ion channel species within ID nanodomains. Thus, ID-localized ion channels clearly merit further investigation, with particular emphasis on their protein neighbors and local structural environment, both within the cell and without. In this regard, available observations on the composition and subcellular location of Na_V_1.5-containing macromolecular complexes hint at significant complexity. Na_V_1.5 has been demonstrated to associate with ankyrin-G (Makara et al., 2014), SAP97 (Petitprez et al., 2011; Gillet et al., 2015), K_ir_2.1 and Kir2.2 (Milstein et al., 2012; Matamoros et al., 2016; Ponce-Balbuena et al., 2018), syntrophin and dystrophin (Petitprez et al., 2011), and CASK (Eichel et al., 2016). Both ankyrin-G and SAP97 preferentially associate with ID-localized Na_V_1.5. However, loss of ankyrin-G reduced functional Na_V_1.5 expression (Makara et al., 2014) whereas loss of SAP97 did not (Gillet et al., 2015). Similarly, Na_V_1.5 at the lateral sarcolemma associates with syntrophin, dystrophin, and CASK. Loss of dystrophin reduced Na_V_1.5 functional expression at this location (Petitprez et al., 2011). Conversely, loss of CASK increased Na_V_1.5 surface expression at the lateral sarcolemma (Eichel et al., 2016). These intriguing findings suggest that we have much to learn about the organization and regulation of sodium channel-rich nanodomains, and the functional implications thereof.

### Pathophysiology

Pathological alterations in Na_V_1.5 function are a well-established cause of cardiac arrhythmias (Veerman et al., 2015; Sottas and Abriel, 2016). Likewise, GJ remodeling is widely recognized as a feature of multiple cardiac pathologies (Jongsma and Wilders, 2000; Stroemlund et al., 2015), and implicated as a contributor to arrhythmogenesis (De Vuyst et al., 2011; Ongstad et al., 2013). While the arrhythmogenic impacts of functional loss of I_Na_ and GJ coupling have long been subjects of intense inquiry, we are only beginning to recognize the effects of nanoscale organization of Na_V_1.5 and Cx43, various scaffolding proteins associated with them, and changes in their ultrastructural environment. An interesting development in this context is the growing appreciation of the non-channel functions of Cx43 and their role in pathophysiology (Agullo-Pascual and Delmar, 2012). Knockdown of Cx43 in mice was associated with concomitant reductions in Na_V_1.5 expression, and I_Na_ density (Jansen et al., 2012) but not with alterations in adherens junctions or desmosomes (Gutstein et al., 2003). Proximity ligation assays indicate that the association between Cx43, and Na_V_1.5 is concentrated within the perinexus (Rhett et al., 2012). This raises the possibility that the aforementioned loss of I_Na_ function may result from the loss of Cx43 to scaffold Na_V_1.5 within perinexal nanodomains, and thereby, preferentially impact these sites. Thus, nanopathy may underlie arrhythmias in the wide array of cardiac pathologies where Cx43 is remodeled (Akar et al., 2007; Desplantez et al., 2007; Dhein et al., 2011; Chkourko et al., 2012). Along similar lines, missense mutations in the desmosomal protein plakophilin-2 were demonstrated to reduce N-cadherin-associated Na_V_1.5 density at the ID, reduce I_Na_, and result in a Brugada syndrome phenotype (Cerrone et al., 2014). Taken together, these results underscore the clinical relevance of understanding the molecular organization of functional nanodomains, and the specific role of nanopathy in disease.

In addition to direct alterations of Na_V_1.5 and/or its molecular partners, nanopathy could also result from pathological changes in nanodomain ultrastructure. In this context, the previously discussed effects of interstitial edema on the perinexal nanodomain take on interesting implications given the wide array of pathologies that are associated with both cardiac edema and arrhythmias (Mehlhorn et al., 1996, 2001; Boyle et al., 2007; Fernandez-Jimenez et al., 2015; Migliore et al., 2015). Additionally, in long QT syndrome type 3 (LQT3; a disorder stemming from pathological gain of Na_V_1.5 function), the Poelzing group recently demonstrated that transient depletion of extracellular Na^+^ within perinexal nanodomains may mitigate risk of premature beats, and that perinexal widening may unmask the latent arrhythmia risk (Greer-Short et al., 2017). More recently, they have demonstrated that wider perinexi associated with the occurrence of AF in human patients (Raisch et al., 2018). While these are early days yet, these results collectively support the view that ultrastructural alterations within Na_V_1.5-rich nanodomains may be a key determinant of arrhythmia risk, and therefore, a potential target for antiarrhythmic therapy.

## The Nano-Machinery of Cardiac Excitation-Contraction Coupling

Our understanding of excitation–contraction coupling (ECC) has followed a similar trajectory to the one outlined for the electrical excitation. Early studies first identified invaginations in the membrane called transverse (T)-tubules (Lindner, 1957) and demonstrated that an activating current localized to those domains prompts contraction (Huxley and Taylor, 1955, 1958). Subsequent work demonstrated that Ca^2+^ entry through the sarcolemma, via L-type Ca^2+^ channels (LTCC), results in calcium-induced calcium release from the sarcoplasmic reticulum (SR) via ryanodine receptor channels (RyR2) to couple electrical excitation with mechanical contraction (Fabiato and Fabiato, 1977). Examination of the structural underpinnings of cardiac ECC led to the identification of a restricted space dubbed the “fuzzy” dyadic space (Lederer et al., 1990) where the sarcolemma and the terminal cisternae of the SR are separated by only ∼12 nm (Forbes and Sperelakis, 1983) (**Figure 1**). These results led to the realization that local protein function and electrochemical fluxes within nanodomains govern ECC rather than bulk effects across larger scales. Indeed, both systolic and diastolic Ca^2+^ concentrations in the dyadic cleft have been found to exceed that of the bulk cytosol (Despa et al., 2014; Popescu et al., 2016), and differences in Na^+^ concentrations have been reported as well (Despa and Bers, 2003).

An early clue to the complexity of the dyadic nanodomain function came with the demonstration by Leblanc and Hume that Na^+^ influx may regulate Ca^2+^ release (Leblanc and Hume, 1990). Specifically, they provided evidence that Na^+^ influx during the action potential upstroke was linked with Ca^2+^ cycling through the action of the Na^+^–Ca^2+^ exchanger (NCX). Interestingly, a component of I_Na_ persists during the plateau phases, dubbed late I_Na_, and is tetrodotoxin (TTX) – sensitive (Conforti et al., 1993). Further investigation along these intriguing lines by the Bridge and Goldhaber groups suggested the involvement of neuronal Na^+^ channel (nNa_V_) isoforms (Torres et al., 2010). This findings are consistent with aforementioned structural results: (1) Na_V_1.5 localizes to the ID and the lateral sarcolemma and (2) nNa_V_s localize to t-tubules (Dhar Malhotra et al., 2001; Maier et al., 2002, 2004; Westenbroek et al., 2013; Radwański et al., 2015) (**Figure 1**). Indeed, up to 50% of the late I_Na_ in canine cardiac myocytes has been reported to be TTX-sensitive (Biet et al., 2012). These results raised the possibility that nNa_V_s, and NCX may constitute essential components of dyadic nanodomains along with LTCCs and RYR2. Since then, multiple groups have provided evidence for co-compartmentation of Na^+^ and Ca^2+^ handling proteins within dyadic nanodomains (Scriven et al., 2000; Jayasinghe et al., 2009). In recent years, evidence has mounted that the high gain system of cardiac Ca^2+^ cycling is tightly regulated by nNa_V_s, and NCX localized within the cleft (Radwański and Poelzing, 2011; Radwański et al., 2013, 2015, 2016; Veeraraghavan et al., 2017). However, the molecular stoichiometry of Na^+^/Ca^2+^ handling machinery (NCX, RyR, Na_V_s) and inter-species differences remain to be elucidated.

An additional layer of complexity in dyadic nanodomain function derives from its structure. It encompasses three spatial compartments – the extracellular space within the t-tubule, the cytosolic subspace, and the terminal cisternae of the junctional SR. In contrast, Na^+^ channel-rich nanodomains at the ID only consist of two compartments, extracellular and intracellular. While much of the inquiry into dyadic nanodomain function has focused on dynamics within the cytosolic subspace, emerging evidence is drawing attention to the complexity of the t-tubular network. Electron microscopy has revealed microfolds along t-tubules (Lavorato et al., 2015), thought to be generated by the action of the protein Bridging integrator 1 (BIN1) (Hong et al., 2014; Fu and Hong, 2016). Experimental observations also suggest that diffusion within t-tubules is significantly slowed in comparison to bulk interstitial space (Blatter and Niggli, 1998; Shepherd and McDonough, 1998; Pasek et al., 2006; Swift et al., 2006). The Lopatin group recently demonstrated that the presence of expansions and constrictions along t-tubules, the result of microfolds, likely accounts for this slow diffusion (Uchida and Lopatin, 2018). This slowed diffusion within t-tubules enables the development of local electrochemical transients within t-tubular nanodomains, paralleling ID nanodomains. This phenomenon has important consequences for cardiac electrophysiology as well as ECC. Likewise local dynamics in luminal Ca^2+^ within the junctional SR would strongly modulate dyadic nanodomain behavior (particularly RYR2 gating) and merit further investigation (Gyorke et al., 2017). While there has been much progress in understanding dyadic nanodomains as the structural and functional units of cardiac ECC, further investigation is needed to understand how structure–function relationships at the nanoscale determine physiology at cellular, tissue, and organ levels.

### Pathophysiology

Dysregulated Ca^2+^ cycling is widely recognized as the cause of arrhythmias in multiple pathological states. Aberrant Ca^2+^ release from the SR can be prompted by SR Ca^2+^ overload, RyR2 dysfunction, or mistimed Ca^2+^ entry across the sarcolemma. This in turn can activate NCX, depolarizing the membrane, and prompting premature beats that can trigger arrhythmias (Berlin et al., 1989; Venetucci et al., 2008). Intriguingly, a recent report suggests that aberrant cytosolic Ca^2+^ levels may also produce arrhythmogenic conditions by facilitating untimely Na_V_ re-opening (Johnson et al., 2018). Overall, the behavior of individual Na^+^ and Ca^2+^ -cycling proteins is fairly well-characterized, yet the interplay between Na^+^ and Ca^2+^ within dyadic nanodomains is very much the subject of active inquiry.

Modeling studies suggest that enhanced late Na^+^ entry into dyadic nanodomains can prompt reversal of NCX, resulting in Ca^2+^ entry into the subspace, and thereby, contribute to arrhythmogenic aberrance Ca^2+^ release (Armoundas et al., 2003; Radwański and Poelzing, 2011). Late I_Na_ carried by nNa_V_s has been implicated in inherited arrhythmia disorders such as catecholaminergic polymorphic ventricular tachycardia (CPVT) (Radwański et al., 2015, 2016). Specifically, Ca^2+^/calmodulin – dependent kinase II (CaMKII) -dependent enhancement of nNa_V_ activity during β-adrenergic stimulation contributes to diastolic Ca^2+^ release and consequent arrhythmias *in vivo* via an NCX-mediated mechanism. Additionally, this pro-arrhythmic mechanism was consistent regardless of whether the CPVT resulted from “leaky” RyRs or from SR Ca^2+^ overload. In the broader context, these results point to pathological overload of cytosolic Na^+^ and Ca^2+^ being inextricably linked, particularly within the dyadic subspace.

Consistent with this hypothesis, aberrant Ca^2+^ release is the principal mechanism of arrhythmogenesis in disorders characterized by pathological gain of Na^+^ channel function. Multiple studies have linked pathological enhancement of nNa_V_ function with arrhythmogenic diastolic Ca^2+^ release. These include mice lacking the Na^+^ channel auxiliary subunit β1 (SCN1B) which exhibit enhanced Na_V_1.3 expression (Lin et al., 2014), a rat pilocarpine-induced status epilepticus model where Na_V_1.1 expression is elevated (Biet et al., 2015), and a murine model of epileptic encephalopathy resulting from a gain of function mutation in Na_V_1.6 (Frasier et al., 2016). While these cases involve direct enhancement of Na^+^ entry into the dyadic nanodomain via nNa_V_s, LQT3 is characterized by gain of Na_V_1.5 function, resulting in global Na^+^ overload throughout the myocyte. Nonetheless, recent evidence indicates that Na^+^ entry into the dyadic subspace via nNa_V_s is still a key element of arrhythmogenesis in LQT3 (Koleske et al., 2018). Paralleling the aforementioned results in inherited arrhythmic syndromes, are studies implicating pathological enhancement of late I_Na_ in arrhythmias in acquired forms of ECC dysfunction such as heart failure (Valdivia et al., 2005; Undrovinas and Maltsev, 2008; Undrovinas et al., 2010; Sossalla and Maier, 2012; Antzelevitch et al., 2014; Makielski, 2016). Specifically, augmentation of Na^+^ influx via Na_V_1.1 (Mishra et al., 2014) as well as of NCX function (Pogwizd and Bers, 2002) in failing hearts have been shown to contribute to arrhythmias.

Given that late I_Na_ is central to aberrant Ca^2+^ cycling and arrhythmogenesis in multiple pathologies, it should come as no surprise that late I_Na_ inhibition by drugs such as ranolazine has demonstrated efficacy as an antiarrhythmic therapy (Burashnikov, 2017). However, emerging research suggests that selective targeting of nNa_V_-mediated Na^+^ entry into dyadic nanodomains may hold even greater promise (Radwański et al., 2015, 2016; Koleske et al., 2018). Additional impetus for pursuing this strategy comes from the dire negative consequences resulting from the off-target effects of non-isoform-selective Na^+^ channel inhibition: although non-selective I_Na_ inhibition suppressed triggered activity following myocardial infarction (The Cardiac Arrhythmia Pilot Study, 1986), the concomitant reduction in excitability precipitated reentrant arrhythmias, thereby increasing mortality (Echt et al., 1991; Starmer et al., 1991). Selective inhibition of nNa_V_ would reduce Na^+^ entry into dyadic nanodomains, thereby ameliorating triggered arrhythmia incidence, without any attendant adverse impact on excitability. Indeed, selective inhibition of nNa_V_s, and of Na_V_1.6 in particular, whether using TTX analogs or a clinically relevant drug, riluzole, has been demonstrated to effectively suppress arrhythmias in murine models of CPVT (Radwański et al., 2015, 2016) and LQT3 (Radwański et al., 2013; Koleske et al., 2018).

Based on this logic, and available evidence, we posit the following requirements for arrhythmogenesis in pathologies directly driven by Ca^2+^ cycling defects (i.e., CPVT), and those arising from QT prolongation: (1) abnormal RyR2 function, whether genetic or acquired, (2) increased dyadic subspace Ca^2+^ levels, and (3) augmentation of nanodomain Na^+^ entry via nNa_V_s (Radwański et al., 2010, 2016; Cheng et al., 2011; Radwański and Poelzing, 2011; Terentyev et al., 2014). Any individual factor in the absence of the other two is unlikely to cause arrhythmia. For instance, genetic defects in the RyR2 complex alone are insufficient to induce triggered activity (Rios and Györke, 2009; Radwański et al., 2016); augmentation of Na^+^ entry via nNa_V_, and SR Ca^2+^ load, secondary to β-adrenergic receptor stimulation, is necessary for arrhythmogenesis (Radwański et al., 2016). On the other hand, LQT may promote CaMKII activity, which in turn modifies the components of the dyadic nanodomain (Terentyev et al., 2014; Viatchenko-Karpinski et al., 2014; Radwański et al., 2016). Given that augmentation of nNa_V_-mediated Na^+^ influx into dyadic nanodomains is a common element of both arrhythmogenic processes, it follows that late I_Na_ inhibition should prove antiarrhythmic in a broad range of pathologies including CPVT (Radwański et al., 2015, 2016), LQT3 (Koleske et al., 2018), LQT type 7 (Andersen-Tawil Syndrome; resulting from loss of repolarization reserve) (Radwański et al., 2013; Janson et al., 2014), and LQT type 8 (Timothy syndrome; resulting from pathological gain of LTCC function) (Gao et al., 2013).

While Na_V_ isoform-selectivity is one focus in developing novel antiarrhythmic drugs, we note that the pharmacological mode of action (i.e., use-dependence vs. tonic block) will also determine therapeutic success. Use-dependent Na_V_ inhibitors can effectively ameliorate triggered arrhythmias (The Cardiac Arrhythmia Pilot Study, 1986). However, under conditions such as elevated heart rates, they can suppress excitability and thereby, exacerbate conduction slowing. This, in turn, precipitates reentrant arrhythmias and increases mortality (Echt et al., 1991; Starmer et al., 1991). In contrast, tonic Na_V_ inhibitors function independently of heart rate, thereby avoiding this adverse effect. Therefore, we postulate that tonic blockade may hold greater potential for delivering efficacy with safety. Intriguingly, a recent report by Buyan et al. (2018) provides clues to fundamental properties (inhibitor protonation state determines binding site) that may lead to development of novel Na_V_ blockers with tailored modes of action. This highlights the need for mechanistically-driven drug development research grounded in understanding of atomic level Na_V_ structure.

As with ID nanodomains, the behavior of dyadic nanodomains is determined not only by the function of their Na^+^/Ca^2+^ cycling protein constituents but also by their local ultrastructure. An example of this is found in failing hearts where β-adrenergic stimulation fails to effectively enhance ECC, despite augmented nNa_V_ and NCX function (Viatchenko-Karpinski et al., 2005). This is likely a result of t-tubules being severely disrupted in failing hearts (Li et al., 2015), compromising cell-wide coordination of individual nanodomains. In addition, the remaining t-tubules in these hearts contain abnormal dyadic nanodomains consisting of nNa_V_s, NCX, and hypersensitized RyRs which are prone to arrhythmogenic aberrant Ca^2+^ release (Belevych et al., 2012). Additionally, loss of BIN1 has been linked to compromised LTCC trafficking as well as loss of t-tubule microfolds in heart failure (Caldwell et al., 2014; Hong et al., 2014; Laury-Kleintop et al., 2015). Both experimental and modeling studies suggest that the latter effect could compromise the previously discussed slowing of diffusion within t-tubules, thereby dysregulating electrophysiology and ECC. Indeed, the Sachse and Bridge groups have demonstrated that the arrhythmia burden in failing hearts is reduced secondary to restoration of the t-tubule network following cardiac resynchronization therapy (Sachse et al., 2012; Lichter et al., 2014; Li et al., 2015). Thus, available evidence points to dyadic nanodomains as promising targets for the prevention of arrhythmias resulting from aberrant Ca^2+^ cycling.

## Conclusion

Current research, propelled by emerging technologies capable of assessing structure/function at the nanoscale, suggests that nanodomains located at the ID and the t-tubule may respectively constitute the functional units of cardiac electrical excitation and ECC. Thus, we are beginning to appreciate nanodomain dysfunction, i.e., nanopathy, as a key mechanistic driver of cardiac disease and arrhythmogenesis. It follows therefore that antiarrhythmic treatments should be designed to correct underlying nanopathies, and indeed, such therapies currently under investigation show a great deal of promise. In the broader context, the emerging understanding of how nanoscale biophysics and biochemistry determine cardiovascular physiology and pathophysiology across protein, cell, tissue, and organ scales may represent a paradigm shift on par with the advent of molecular biology. It should drive multiple avenues of scientific and medical research including (1) new diagnostic approaches that can non-invasively interrogate functional nanodomains within patients, (2) new methods to assess nanodomain alterations during autopsies, and (3) new therapeutic approaches designed to restore nanodomain structure/function.

## Author Contributions

RV, CNJ, SG, and PR drafted the work or revised it critically for important intellectual content.

## Conflict of Interest Statement

The authors declare that the research was conducted in the absence of any commercial or financial relationships that could be construed as a potential conflict of interest.
